# EMT Transcription Factors Are Involved in the Altered Cell Adhesion under Simulated Microgravity Effect or Overloading by Regulation of E-cadherin

**DOI:** 10.3390/ijms21041349

**Published:** 2020-02-17

**Authors:** Shuliang Shi, Qiao Li, Qiuying Cao, Yan Diao, Yao Zhang, Lei Yue, Lijun Wei

**Affiliations:** 1 School of Life Science and Technology, Harbin Institute of Technology, Harbin 150001, China; liangss@hit.edu.cn (S.S.); lijingqiong313127@163.com (Q.L.); xinyitian89@126.com (Q.C.); zhangyao83326@163.com (Y.Z.); yuelei@hit.edu.cn (L.Y.); 2 College of Life Science, China West Normal University, Nanchong 637002, China; 3 State Key Laboratory of Space Medicine Fundamentals and Application, Chinese Astronaut Research and Training Center, Beijing 100094, China

**Keywords:** simulated microgravity effect (SMG), mechanical overloading (OL), cell adhesion, HUVEC and MCF-7 cell

## Abstract

In order to study the effect of stress changes on cell adhesion, HUVEC, and MCF-7 cells were treated with simulated microgravity effect (SMG) and overloading (OL). Methods: Rotating Wall Vessel (2D-RWVS) bioreactor was used to create different culture conditions. In addition, the alteration of cell adhesion states, adhesion proteins, and relating factors of adhesion molecules under these two conditions were detected using cell adhesion assay, immunofluorescence, western blot, and qRT-PCR technology. Results: The results showed that the adhesion of cells decreased under SMG, while increased under OL. The expressions of integrin β1, paxillin, and E-cadherin under SMG condition were down-regulated as compared to that of the control group showing a time-dependent pattern of the decreasing. However, under OL condition, the expressions of adhesion proteins were up-regulated as compared to that of the control group, with a time-dependent pattern of increasing. EMT transcription factors Snail, twist, and ZEB1 were up-regulated under SMG while down-regulated under OL. Conclusion: Collectively our results indicated that cells could respond to stress changes to regulate the expressions of adhesion proteins and adapt their adhesion state to the altered mechanical environment. The altered cell adhesion in response to the mechanical stress may involve the changed expression of EMT-inducing factors, Snail, Twist, and ZEB1under the SMG/OL conditions.

## 1. Introduction

The space environment has the characteristic of high vacuum, microgravity, heavy radiation, changing magnetic field, extreme temperature, and other conditions, which will damage the organisms entering it. During space flight, gravity acceleration changes in different flight stages. When the spacecraft accelerates to rise up or slow down and landing to the surface of the Earth, an acceleration of 3–5× *g* may occur, which is so-called hypergravity. While during spacecraft flying in orbit, gravity acts as a centripetal force, and objects show weightlessness or microgravity. Microgravity can cause damage to the heart, brain, bones, muscles, etc., such as bone loss, muscle atrophy [1,2,3], etc. While hypergravity can cause changes in systemic blood distribution, visual impairment, brain dysfunction, difficulty breathing, chest pain, and severe tearing of internal organs [4,5].

Changes in mechanical stress can cause structural and functional alteration of cells, including cell cycle arrest at G2/M, inhibition of cell proliferation, multipolar spindle formation in metaphase, and enhanced apoptosis [6]. At present, some commonly recognized biomechanical signals to chemical signaling pathways in domestic and foreign research include integrins and Rho family [7].

Cell adhesion molecules are a class of molecules that mediate contact and binding between cells or between cells and extracellular matrix. Most of them belong to glycoproteins and function through the formation of receptor-ligand binding. The expression of cell adhesion proteins directly reflects the degree of adhesion between cells, or between cells and extracellular matrix [8]. Integrin is a heterodimeric transmembrane receptor composed of two subunits, α and β. These two subunits are combined by non-covalent bonds, with combinations of at least 24 types [9]. Alam’s research indicates that integrin molecules can bind to different extracellular matrix (ECM) molecules due to overlapping affinities [10]. The relationship between cytoplasmic matrix and cell signaling is the result of the dynamic interaction of integrins with proteoglycans and growth factor receptors. ECM consists of glycoproteins, collagen, and proteoglycans that form a dynamic microenvironment [11]. The ECM plays an important role in maintaining the shape and rigidity of some tissues, and the cells can grow only when the ECM is attached to the cell surface. The ECM not only supports and maintains the morphology of the cells but also provides a microenvironment for the cells to grow and migrate [12].

Paxillin involved in the ECM. There is an actin-membrane attached cytoskeletal protein at the site of cell adhesion, which is mainly present in the local adhesion zone [13]. Paxillin is major component of focal adhesion with a molecular weight of 68 kDa regulating cytoskeletal reconstruction and migration of cells through phosphorylation of the tyrosine residue of the paxillin [14,15,16].

As a calcium-dependent transmembrane glycoprotein, E-cadherin mediates and maintains the formation of adherence junctions between neighboring homologous cells. The trans-interaction of E-cadherin between the extracellular EC domain of E-cadherin on neighboring cells is critical for the formation of adherence junctions. The intracellular part of E-cadherin can connect with the actin cytoskeleton through p120-catenin and β-catenin [17]. This adherence junction not only connects adjacent cells together to maintain morphology and polarity of epithelial cells but also participate in signal transduction between and within cells [18,19]. The binding of α-catenin and β-catenin to the cytoplasmic domain of E-cadherin enhanced the cell adhesion activity [20]. Afadin is an actin-binding protein that mediates the interaction of adherence junction with actin skeleton [21].

Our previous experiments have shown that the number of cells decreased after SMG treatment [6], so it was speculated that cell abscission was related to the decrease of cell adhesion. Therefore, the cell culture models with different mechanical conditions were designed to clarify the correlations between stress changes with cell exfoliation and cell adhesion. In this study, we found that the adhesion of HUVEC and MCF-7 cells were significantly changed under the conditions of SMG and overloading. So we continue to study whether the expression of adhesion proteins in HUVEC and MCF-7 cells also changes accordingly. We found that mechanical stress can induce the change of expression of adhesion proteins, which are consistent with the alteration of cell adhesion. To address the mechanism of the altered expression of E-cadherin in SMG or OL conditions, the RNA expressions of Snail, Twist and ZEB1 were determined by qRT-PCR after the simulated microgravity effect or overloading treatments.

This study shows that the change of stress can affect cell adhesion, include the cell-to-cell, cell-to-matrix adhesion. Simulated microgravity effect decreases cell adhesion, and overloading increases cell adhesion. Snail, Twist, and ZEB1 are involved in the down/up-regulation of E-cadherin during the SMG/OL.

## 2. Results

### 2.1. Cell Exfoliation Rate Analysis after Mechanical Treatment

**Figure 1:**
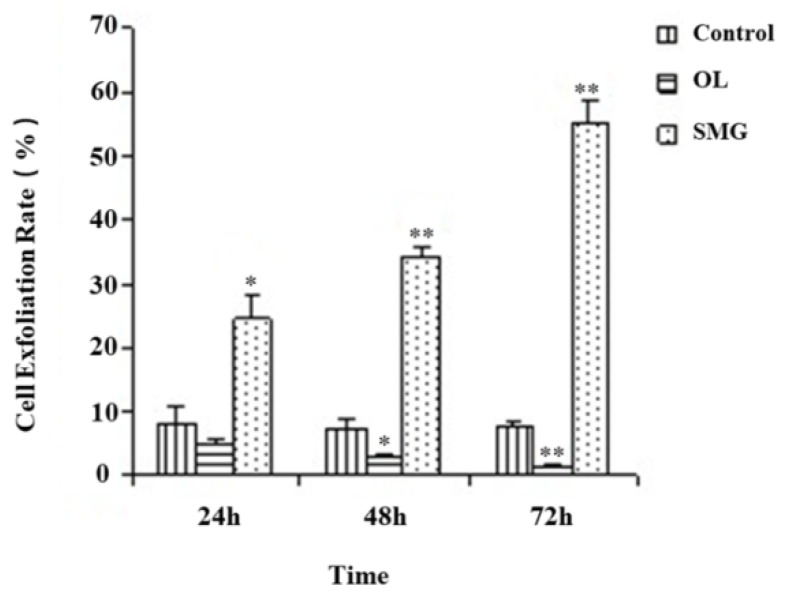
HUVEC cell exfoliation rate after trypsin treatment. * *p* < 0.05, Significant difference; ** *p* < 0.01, extremely significant difference. Cell Exfoliation Rate = Exfoliated cells B/ Total Number A (*n* = 3).

**Figure 2:**
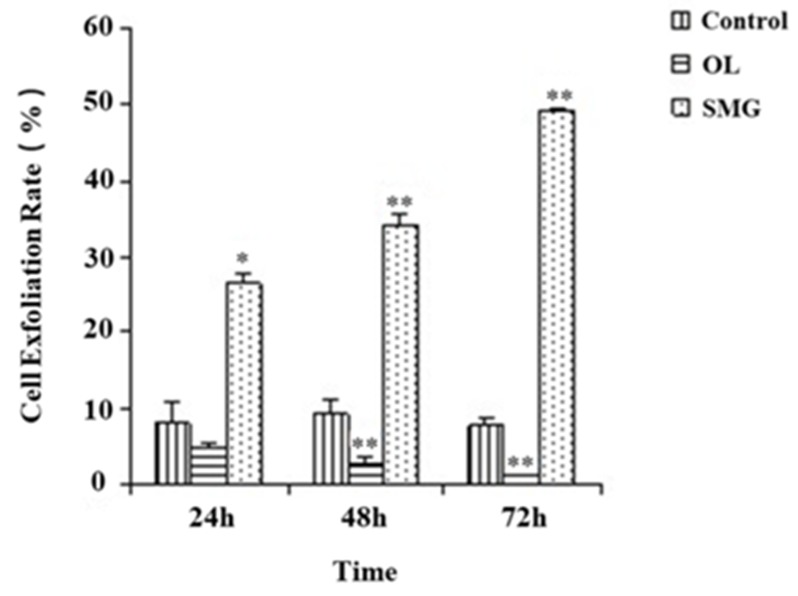
MCF-7 cell exfoliation rate after trypsin treatment. * *p* < 0.05, Significant difference; ** *p* < 0.01, extremely significant difference. Cell Exfoliation Rate = Exfoliated cells B/ Total Number A (*n* = 3).

The exfoliation of HUVEC and MCF-7 cells was counted by trypsin digestion combined with cell count (Tables S1 and S2, and Figure 1 and Figure 2). The correlation was analyzed by the Chi-square test. From Figure 1, we can clearly see that the exfoliation rate of HUVEC cells at 24 h of simulated microgravity effect was significantly different from that of the ground control group (*p* < 0.05), while the exfoliation rate of HUVEC cells at 24 h of force loading was not significantly different from that of the ground control group (*p* > 0.05); at 48 h, the exfoliation rate of HUVEC cells began to show significant difference compared with the ground control group (*p* < 0.05). The results showed that the change of force could affect cell exfoliation. Under the simulated microgravity effect, the cell exfoliation rate increased significantly with the increase of time. On the contrary, in the mechanical loading group, the cell exfoliation rate decreased significantly. At 48 h and 72 h, the change of stress was correlated with the exfoliation rate of cells.

### 2.2. Localization and Semi-Quantitative Analysis of Adhesion Proteins by Immunofluorescence Analysis

HUVEC and MCF-7 cells were cultured in a suitable amount and placed on coverslips. The cells were treated with simulated microgravity effects and mechanical overloading. The expression of integrin β1, paxillin, and E-cadherin were observed by immunofluorescence after 24, 48, and 72 h, and the relative fluorescence intensity was analyzed using Image J software.

#### 2.2.1. Integrin β1 Changed in HUVEC and MCF-7 Cells

**Figure 3:**
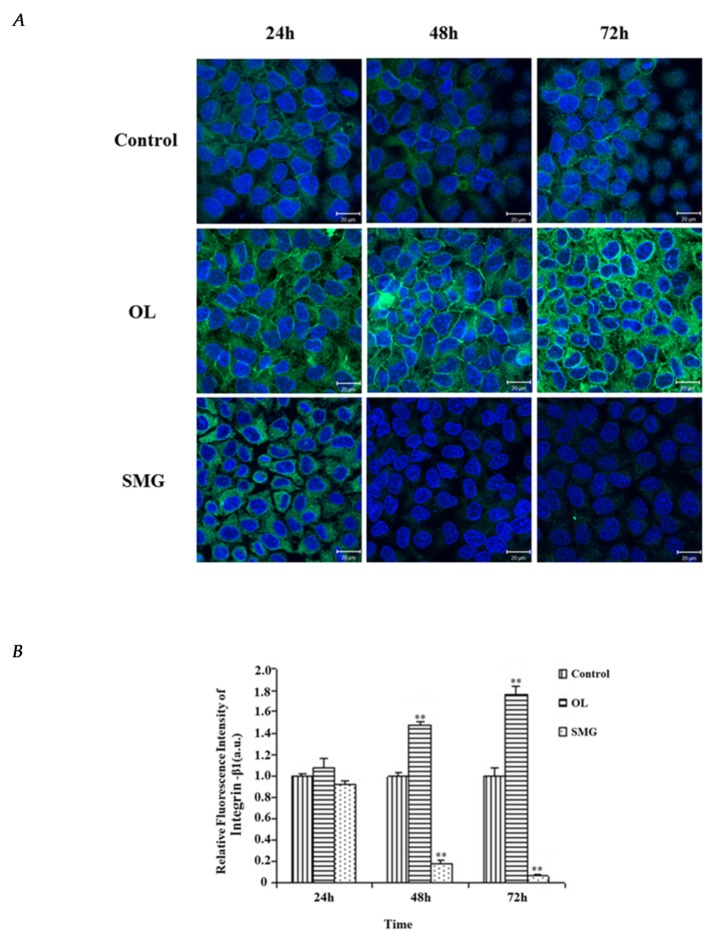
The expression of integrin β1 in HUVEC cells after mechanical treatment. (**A**) The results of immunofluorescence experiments. The blue color indicates the nucleus, and the green color represents the integrin β1 protein. a. 24 h Control; b. 48 h Control; c. 72 h Control; d. 24 h Overload; e. 48 h Overload; f. 72 h Overload; g. 24 h SMG; h. 48 h SMG; i. 72 h SMG. (**B**) Relative fluorescence intensity of integrin β1 in HUVEC cells. significant difference; ** *p* < 0.01, extremely significant.

The mechanically treated HUVEC cells were treated consistently in both staining and photographing. The results showed that the expression of integrin β1 in HUVEC cells was low under normal gravity, but high under force loading, and the location of integrin β1 expression was distributed between cells, while under simulated microgravity effect, the expression of integrin β1 was almost non-existent (Figure 3A).

ImageJ analysis (Figure 3B) showed that the fluorescence intensity of integrin β1 in the ground control group remained basically unchanged at 24, 48, and 72 h. The fluorescence intensity of integrin β1 under the simulated microgravity effect was not significantly different from that of the ground control group at 24 h (*p* > 0.05). At 48 h, the fluorescence intensity of integrin β1 was significantly different from that of the ground control group (*p* < 0.01). The trend of 72 h was the same as that of 48 h (*p* < 0.01). Under the condition of overloading, the fluorescence intensity of integrin β 1 was not significantly different from that of ground control at 24 h (*p* > 0.05). At 48 h, the fluorescence intensity of integrin β 1 was significantly different from that of ground control (*p* < 0.01). At 72 h, the fluorescence intensity of integrin β 1 was also significantly different from that of the ground control group (*p* < 0.01). And we can also see that the fluorescence intensity of integrin β1 decreases with time under simulated microgravity effect, while the fluorescence intensity of integrin β1 increases with time under overloading conditions.

**Figure 4:**
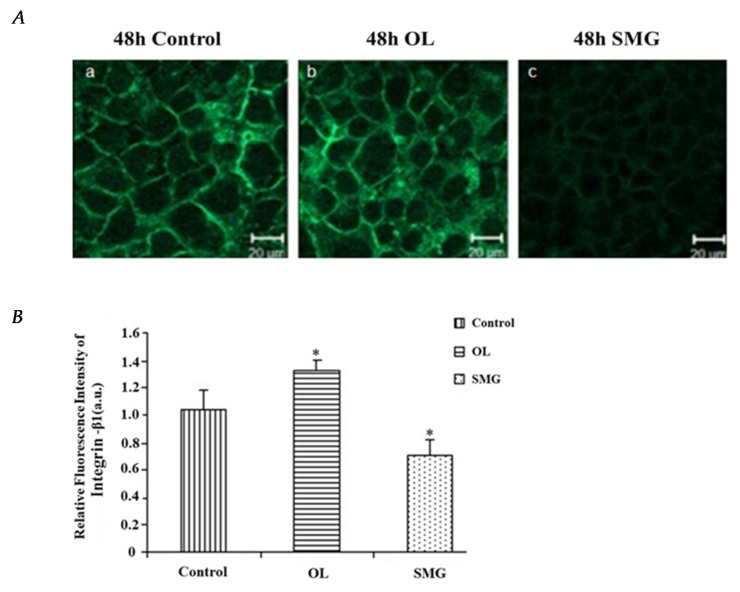
The expression of integrin β1 in MCF-7 cells after mechanical treatment. (**A**) The results of immunofluorescence experiments. a. 48 h Control; b. 48 h Overload; c. 48 h SMG. (**B**) Relative fluorescence intensity of integrin β1 in MCF-7 cells after mechanical changes. * *p* < 0.05, significant difference; extremely significant.

The fluorescence intensity of integrin β1 in MCF-7 is relatively stronger under the overloading condition and relatively weaker under simulated microgravity effect (Figure 4A).

According to the analysis of Image J (Figure 4B), under overloading conditions of 48 h, the fluorescence intensity of integrin β1 in MCF-7 was significantly different from that of ground control group (*p* < 0.05), which was higher than that of simulated microgravity effect, the fluorescence intensity of integrin β1 was significantly different from that of ground control group (*p* < 0.05), which was lower than that of ground control group. It is indicated that the expression of integrin β1 was increased under overloading and decreased under simulated microgravity effect.

#### 2.2.2. Paxillin Changed in HUVEC and MCF-7 Cells

**Figure 5:**
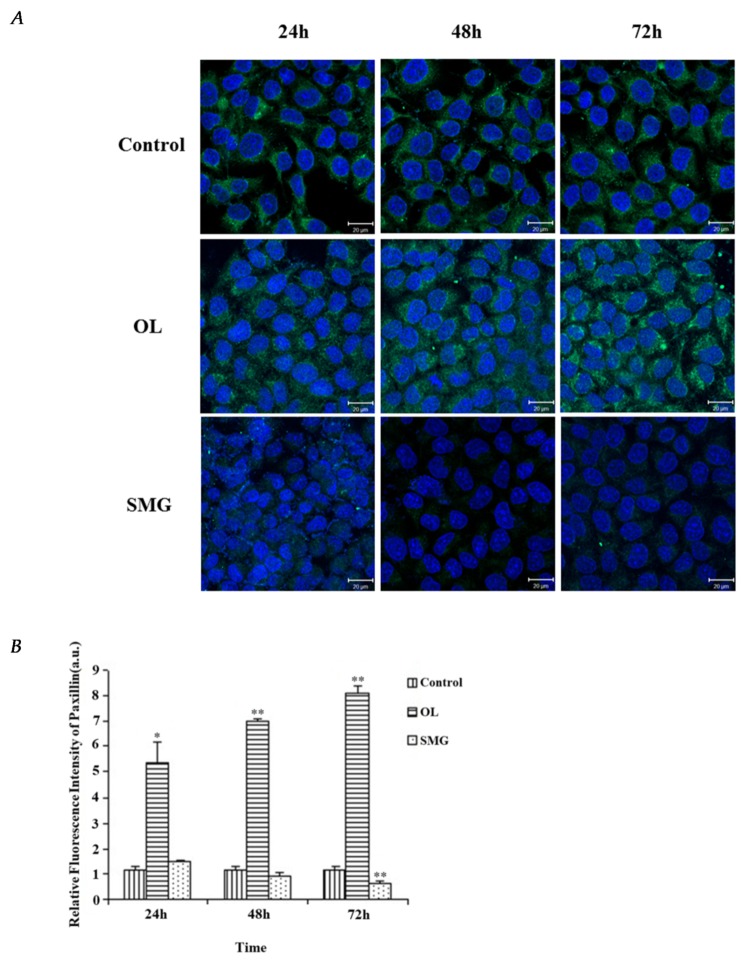
Expression of paxillin in HUVEC cells after cell mechanics treatment. (**A**) The results of immunofluorescence experiments. The blue color indicates the nucleus, and the green color represents the paxillin protein. a. 24 h Control; b. 48 h Control; c. 72 h Control; d. 24 h Overload; e. 48 h Overload; f. 72 h Overload; 24 h SMG; h.48 h SMG; i. 72 h SMG. (**B**) Relative fluorescence intensity of paxillin in HUVEC cells after mechanical changes. * *p* < 0.05, significant difference; ** *p* < 0.01, extremely significant.

It was shown that the paxillin protein was distributed around the nucleus of HUVEC cells clearly under normal gravity conditions. Under overloading conditions, the distribution of paxillin concentrates on the nucleus of HUVEC cells compared with the ground. However, paxillin was hardly expressed under simulated microgravity effect (Figure 5A).

The relative fluorescence intensity was further calculated by Image J (Figure 5B). It can be seen that the fluorescence intensity of paxillin gradually decreased with the increase of microgravity time. At 72 h, the fluorescence intensity of paxillin was significantly different from that of the ground control (*p* < 0.01). Under the condition of overloading, the fluorescence intensity of paxillin increased in 24 h, 48 h, and 72 h, and the difference was very significant compared with that of ground control (*p* < 0.01).

**Figure 6:**
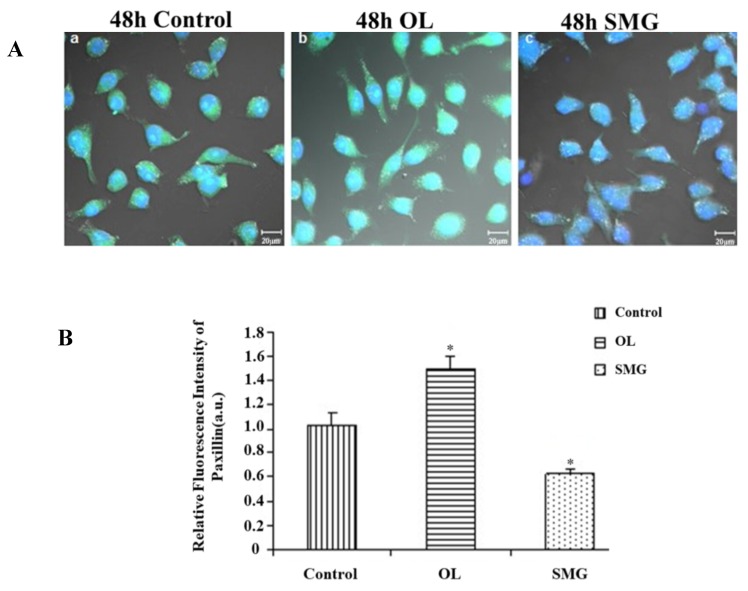
Expression of paxillin in MCF-7 cells after cell mechanics treatment. (**A**) The results of immunofluorescence experiments. The blue color indicates the nucleus, and the green color represents the paxillin protein. a. 48 h Control; b. 48 h Overload; c. 48 h SMG. (**B**) Relative fluorescence intensity of paxillin in MCF-7 cells after mechanical changes. * *p* < 0.05, significant difference; extremely significant.

In MCF-7 cells, under normal gravity conditions, paxillin expressed normally; under the conditions of overloading, the expression of paxillin was high, and its distribution extended along the direction of cytoplasm; while under simulated microgravity effect, paxillin was hardly express (Figure 6A).

Image J analysis showed that under the overloading condition, the fluorescence intensity of paxillin in MCF-7 was significantly different from that of ground control group (*p* < 0.05), and the fluorescence intensity of paxillin was stronger than that of ground control group; under simulated microgravity effect, the fluorescence intensity of paxillin was significantly different from that of ground control group (*p* < 0.05), fluorescence intensity was weaker than that of ground control. Explanatory overload increases the expression of paxillin, and simulated microgravity effect decreases the expression of paxillin (Figure 6B).

#### 2.2.3. E-Cadherin Changed in MCF-7 Cells

E-cadherin is a kind of cadherin, a family of Ca^2+^ depended on gluconin between cells and is closely related to the metastasis of tumors, so we further studied the expression of E-cadherin in MCF-7.

**Figure 7:**
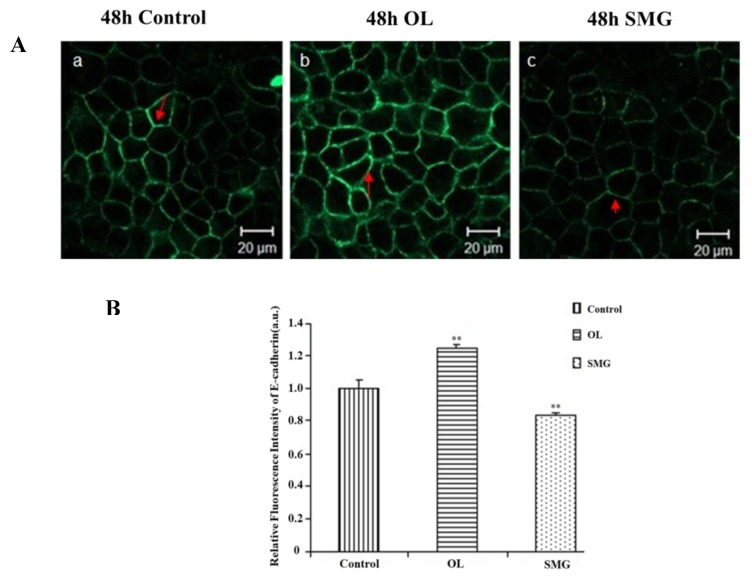
The expression of E-cadherin in MCF-7 cells after mechanical treatment. (**A**) The results of immunofluorescence experiments. The blue color indicates the nucleus, and the green color represents the paxillin protein. a. 48 h Control; b. 48 h Overload; c. 48 h SMG. (**B**) Relative fluorescence intensity of E-cadherin in MCF-7 cells after mechanical changes. Significant difference; ** *p* < 0.01, extremely significant.

The expression of E-cadherin was observed by confocal microscopy in the ground control group, overloading group, and simulated microgravity effect group after treated for 48 h (Figure 7A). Compared with the control group, the expression of E-cadherin was stronger after overloading and weaker after the simulated microgravity effect treatment.

Image J analysis showed that at 48 h, under overloading conditions, the fluorescence intensity of E-cadherin was significantly different from that of the ground control group (*p* < 0.01), and was stronger than that of the ground control group (Figure 7B). Under the simulated microgravity effect, the fluorescence intensity of E-cadherin was significantly different from that of the ground control group (*p* < 0.05), which was weaker than that of the ground control group. The results showed that the overloading increased the expression of E-cadherin, and the simulated microgravity effect decreased the expression of E-cadherin.

### 2.3. Quantitative Analysis of Adhesion Proteins by Western Blot

After 24 h, 48 h, and 72 h of cell culture, the cells were collected, and the total protein was extracted. Before Western Blot, the total protein concentration of each group was determined to adjust the sample size, and the Western blot for integrin β1, paxillin, and E-cadherin were sampled with 40 μg of total protein. Western Blot’s results were analyzed using Quantity One software to determine the effect of force changes on adhesion proteins.

#### 2.3.1. Integrin β1 Expression Analyzed by Western Blot

**Figure 8:**
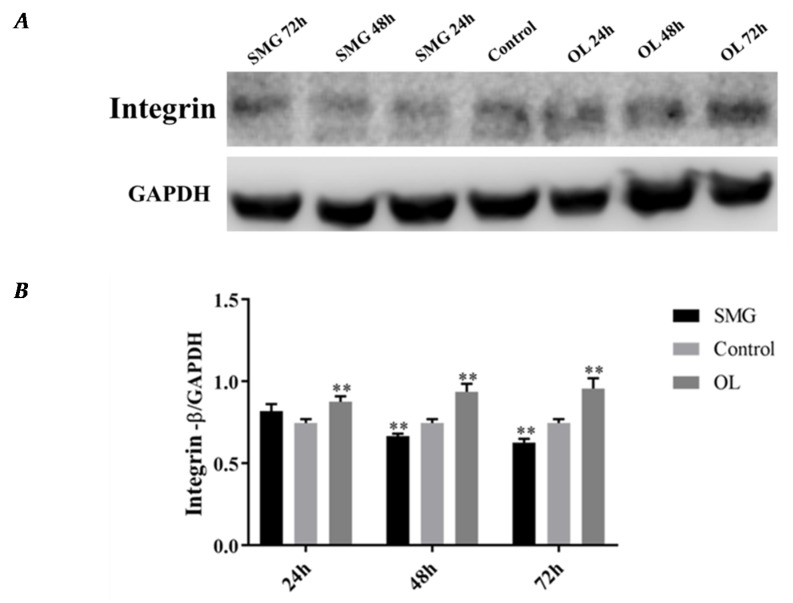
Western Blot analysis of integrin β1 in HUVEC cells after mechanical loading. (**A**) Results for Western blots. (**B**) Reflective densitometry of Western blots. Significant difference; ** *p* < 0.01, extremely significant.

Western Blot results showed that the expression of integrin β1 in HUVEC cells in simulated microgravity effect group had no significant changes, while significantly decreased under 48 h and 72 h SMG. While in overloading group integrin β1 was higher than that of the ground control group, and the expression of integrin β1 increased with time (Figure 8A,B). The protein expression changes have a similar tendency with immunofluorescence analysis.

**Figure 9:**
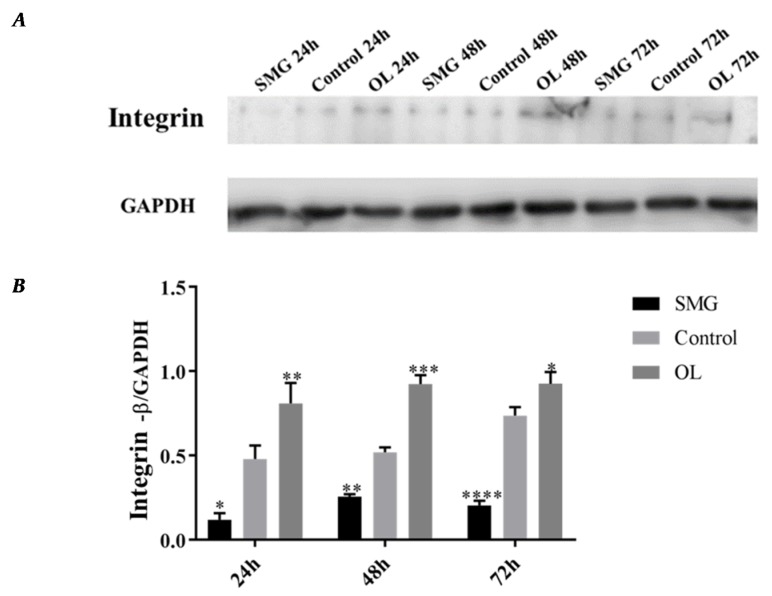
Western blot analysis of integrin β1 in MCF-7 cells after mechanical changes. (**A**) Results for Western blots. (**B**) Reflective densitometry of Western blots. * *p* < 0.05, Significant difference; ** *p* < 0.01, *** *p* < 0.001 and **** *p* < 0.0001, extremely significant difference.

After simulated microgravity effect and mechanical loading, the expression levels of integrin β1 was increased under overloading conditions and decreased in simulated microgravity effect compared with that in the ground control group in MCF-7 cells. Integrin β1 expression changed with time going, increase or decrease more than double (Figure 9A,B). The results were in good agreement with the results of 48 h immunofluorescence analysis.

#### 2.3.2. Paxillin Expression Analyzed by Western Blot

**Figure 10:**
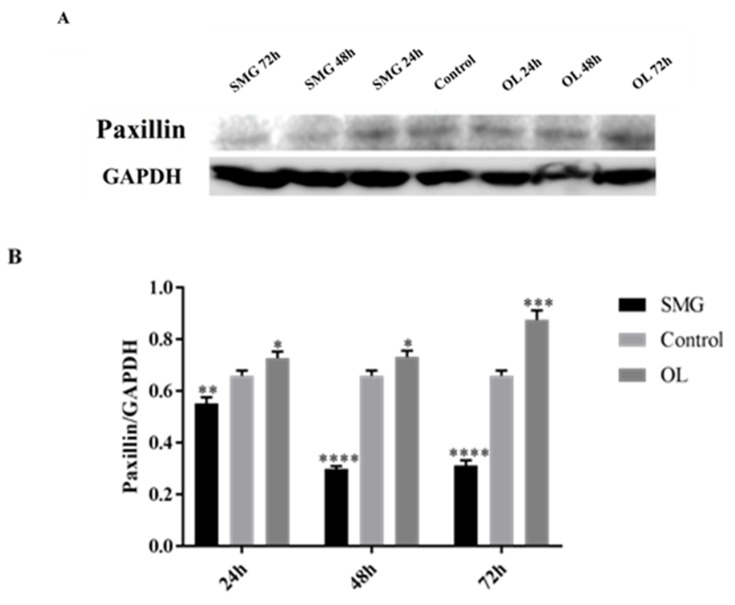
Western blot analysis of cells paxillin in HUVEC cells after mechanical changes. (**A**) Results for Western blots. (**B**) Reflective densitometry of Western blots. * *p* < 0.05, Significant difference; ** *p* < 0.01, *** *p* < 0.001 and **** *p* < 0.0001, extremely significant.

Compared with the ground control, paxillin expression in HUVEC cells also have a similar tendency with integrinβ1 (Figure 10A,B). At 48 and 72 h, the expression of paxillin in the simulated microgravity effect group was half of that in the ground control group. It was shown that the expression of paxillin in HUVEC cells at 24 h, 48 h, and 72 h in the overloading group was higher than that of the ground control group, and the expression of paxillin increased with time.

The previous immunofluorescence has shown that the expression of paxillin in HUVEC cells has changed under the condition of overloading. Quantitative analysis of paxillin expression in HUVEC cells by Western Blot revealed that overloading in HUVEC cells significantly increased the expression of paxillin, further indicating that the mechanical treatment did affect the expression of paxillin in HUVEC cells.

**Figure 11:**
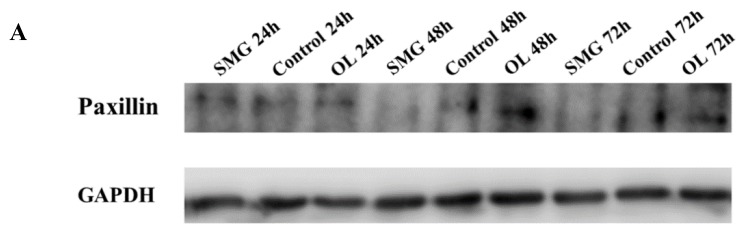

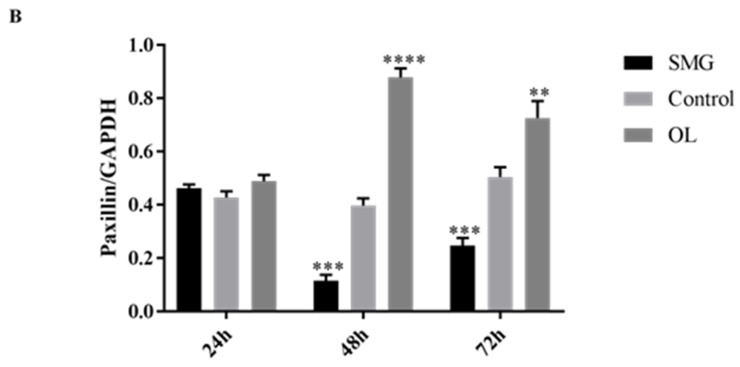
Western blot analysis of cells paxillin in MCF-7 cells after mechanical changes. (**A**) Results for Western blots. (**B**) Reflective densitometry of Western blots. Significant difference; ** *p* < 0.01, *** *p* < 0.001 and **** *p* < 0.0001, extremely significant.

The expression of paxillin in MCF-7 showed no difference with the control group under simulated microgravity effect or overloading conditions at 24 h, but have significant difference after treated for 48 and 72 h (Figure 11A,B). The expression of paxillin at 48 h in the SMG group was half that of the control group, and doubled in the OL group. These results were in good agreement with the results of 48 h immunofluorescence analysis.

#### 2.3.3. E-Cadherin Expression Analyzed by Western Blot

**Figure 12:**
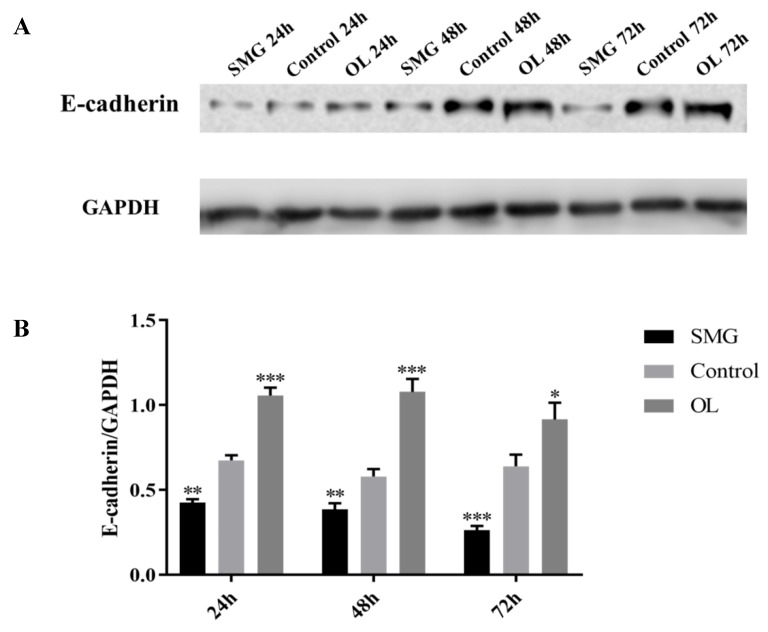
Western blot analysis of E-cadherin in MCF-7 cells after mechanical changes. (**A**) Results for Western blots. (**B**) Reflective densitometry of Western blots. * *p* < 0.05, Significant difference; ** *p* < 0.01 and *** *p* < 0.001extremely significant.

The expression of E-cadherin in MCF-7 cells was similar to the trend of integrin β1 and paxillin (Figure 12A,B). The overall trend is that the expression of the SMG group was lower than that of the control group, while in the OL group, the expression of E-cadherin was significantly higher than control. Significant changes of E-cadherin expression were observed after 24 h treatment. Under 48-h overload, the expression in E-cadherin reached two times of the control, while under 72-h microgravity, it was 0.5 times lower than control. These results were in good agreement with the 48-h immunofluorescence assay.

### 2.4. Snail, Twist and ZEB1 Expression Analyzed by qRT-PCR

**Figure 13:**
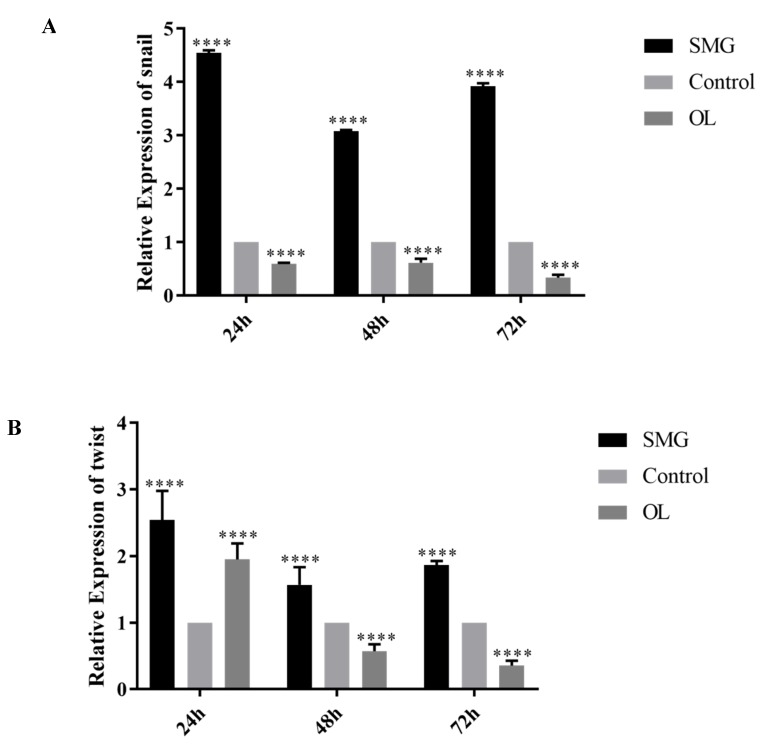

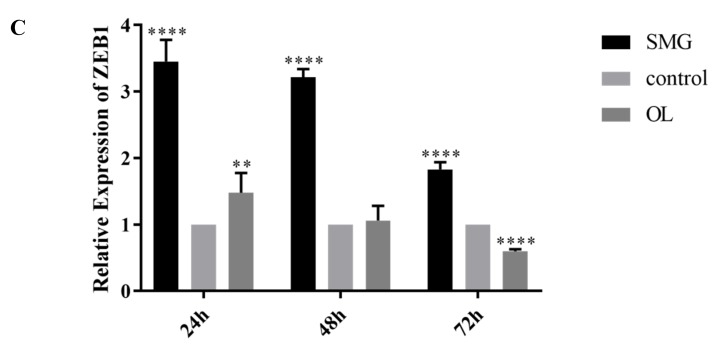
The mRNA expression changes of Snail (**A**), Twist (**B**), and ZEB1(**C**) in MCF-7 cells were detected by qRT-PCR. The statistical results shown represent the means ± SD (*n* = 3). Significant difference; ** *p* < 0.01and **** *p* < 0.0001, extremely significant.

In our experiments, the simulated microgravity effect largely reduced the expression of E-cadherin, while the overloaded condition increased the expression of E-cadherin in MCF-7 cells (Figure 7 and Figure 12). To address the mechanism of the altered expression of E-cadherin in SMG or OL conditions, the RNA expressions of Snail, Twist, and ZEB1 were determined by qRT-PCR after the simulated microgravity effect or overloading treatments (Figure 13A–C).

The expression of Snail, Twist and ZEB1 were all largely increased in 24-, 48-, and 72-h treatment of SMG treatment. While the highest expression of Snail, Twist and ZEB1 were observed in 24-h SMG treatment, all their expression showed a tendency of reduction in 48- and 72-h SMG treatment as compared to their expression level in 24-h SMG treatment. Among them, the Snail expression showed the highest expression level after 24-h SMG treatment (4.5 times higher than control). During the OL condition, the reduced expression of snail was observed in all 24-, 48-, and 72-h OL treatment, as compared to the control condition. On the other hand, the expression of Twist and ZEB1 were increased at 24-h OL treatment, while reduced gradually at 48- and 72-h OL treatment. Previously investigated in breast cancer and our results suggested the possible involvement of Snail, Twist, and ZEB1 in the down-regulation of E-cadherin during the SMG [22]. As the time point of increased expression of Snail was consistent with the down-regulation of E-cadherin at 24-h SMG treatment, the increased expression of Snail mainly was correlated with the down-regulation of E-cadherin in SMG treatment. However, the roles of Twist and ZEB1 in the down-regulation of E-cadherin during SMG conditions are waiting to be elucidated.

## 3. Discussion

Buken et al. [23] ound simulated microgravity effect using random positioning machine (RPM) could induce changes in the cytoskeleton, ECM, focal adhesion, and growth behavior of NHDF. Tan et al. [24] used clinostat to simulate microgravity and found that SMG inhibited focal adhesions, leading to inhibition of FAK, RhoA, and mTORC1 pathway, which results in activation of the AMPK pathway and reduced melanoma cell proliferation and metastasis. Using a proteomic tool, Sahana et al. [25] found that when MCF7 cells were cultured at RPM, couples of junction-related proteins, including E-cadherin, claudin-3 and integrin-β4 were down-regulated as well as the negative regulators of E-cadherin. Among them, the expression of E-cadherin was down-regulated both in adherent and multicellular spheroids (MCS) subpopulations, with the extent of down-regulation of E-cadherin was extremely higher in MCS than adherent subpopulation and the control cells under 1g condition [25]. Kopp et al. [26]. found that genes involved in cell structure, shape, adhesion, migration, and angiogenesis suggested significant changes after a 10d-RPM-exposure. Under the short-term real microgravity condition, the protein level of E-cadherin was reduced, along with integrin β1 [26]. The elevation of ICAM-1, VCAM-1, and CD44 could be detected after 31st parabola flight. Collectively, although the effect of microgravity on the expression of adhesion molecules has been investigated, the mechanism underlying the down-regulation of adhesion molecules in the SMG or real microgravity condition is still elusive. The mechanisms that the cells sense the mechanistic alteration under the condition of microgravity and overload may involve the functions of similar sets of molecules. To understand mechanistic correlation of adhesion molecules under the condition of SMG and overload, a comparison between the effects of weightlessness and overload on cell adhesion was made using a 2D-RWV model.

In our experiments, we found that the mechanical overloading and simulated microgravity effect have a significant impact on the adhesion of HUVEC and MCF-7 cells. Mechanical overloading improved the adhesion of HUVEC and MCF-7, and simulated microgravity effect reduced the adhesion of HUVEC and MCF-7 on the coverslips. Further, the expression of paxillin and integrin β-1 in HUVEC cells and the expression of paxillin, integrin β-1, and E-cadherin in MCF-7 were studied under overloading or simulated microgravity effect, and the effect of mechanical changes on cell adhesion was explained.

Integrin β1 is expressed on the surface of most cells and mediate adhesion between cells and extracellular matrix [27]. The down expression of integrin β1 could inhibit the adhesion of cells to ECM. Under the simulated microgravity effect, the expression of integrin β-1 decreased, but on the contrary, the expression of it increased under the overloading condition. Because integrin β-1 can be used as a stress receptor [28], when cells are stimulated by external forces, the force will be transmitted to the cells, and converted into biological signals, resulting in changed morphology, adhesion, and rearrangement of cytoskeleton, the functions correlated to rearrangement and expression of cell adhesion factors. For MCF-7 cells, we also can detect the change of integrin β-1 expression, which can explain that mechanical changes can alter cell adhesion. To investigate cell adhesion and the expression of integrin β1 under the condition of SMG in MCF-7 cells, immunofluorescence and Western Blot analysis were used. We found that the expression of integrin β1 was reduced, which was consistent with the decreased cell adhesion, suggesting the correlation between decreased cell adhesion and reduced expression of integrin β1. The interaction of cells with the ECM plays a crucial role in the development and in the tissue cycle. After binding of integrin to ECM, adhesion molecules including paxillin, vinculin, α-actinin, and FAK will be recruited to the cytosolic domain of integrin to form a focal adhesion complex [29]. Then integrin will be activated and clustered to enhance the binding affinity and avidity to ECM. At the cytosolic domain, integrin interacts with the actin filaments mediated by paxillin, vinculin, and α-actinin. Therefore, at the point of contact, focal adhesion complex not only regulate cell-matrix adhesion but also serve as anchors for the actin cytoskeleton and serve as signal transducer from ECM to cells. However, recent evidence suggests that signaling in mechanical stimulation is associated with both microfilaments and vimentin in several endothelial cell types that are rich in αvβ3 integrin contacts [30].

Paxillin is an important factor of focal adhesion complex connecting the mechanical stress from ECM to the actin stress fiber when bound to the intracellular domain of integrin inside the cells. In our experiments, both normal cells (HUVEC) and cancer cells (MCF-7) showed a downward trend in their adhesion under SMG conditions. Previous studies have shown that the down-expression of paxillin resulted in altered cell morphology and decreased cell adhesion [31]. Our results showed that paxillin decreased while HUVEC and MCF-7 cells were treated in SMG condition, while up-regulated under overloading condition from the time point of 24 h to 72 h. The decreased or increased expression of paxillin in MCF-7 cells could also be detected 48h after treatment with SMG or overload, indicating the delayed response of MCF-7 cell to the mechanical stress as compared to HUVEC cells. The altered expression of paxillin under the condition of SMG or overload was well consistent with the previous publication. E-cadherin is a calcium-dependent cell adhesion glycoprotein, which can mediate cell-cell junctions, and maintains cell integrity through interaction with the counterpart on the surface of the neighboring cells [32]. The expression of E-cadherin in MCF-7 cells decreased under the SMG conditions, while increased when MCF-7 cells were treated with overloading conditions. E-cadherin plays a critical role in the establishment and maintenance of cell-cell adhesions and polarity through the formation of adheren junctions in a variety of cell types. The disruption of adheren junctions causes loosening of cell-cell contacts, leading to disorganization of tissue architecture [33]. After simulated microgravity effect and mechanical overloading treatment, it was observed that the expression of E-cadherin in MCF-7 cells was significantly higher under the mechanical overloading condition than that in the ground control group. On the contrary, the expression of E-cadherin in the simulated microgravity effect group was less than that in the ground control group. The altered expression of E-cadherin in MCF-7 cells in response to SMG or overload could result in decreased or enhanced cell adhesion. Our results supported the functional connections between the loss of expression of E-cadherin with reduced cell adhesion and increased expression of E-cadherin with enhanced cell adhesion after MCF-7 cells were treated with SMG or overload (Figure 2, Figure 7, Figure 12).

The expression of E-cadherin is frequently targeted during epithelial-mesenchymal transition (EMT) through down-regulation by transcriptional repressors, including Snail, Twist, ZEB1, and so on [34]. The transcriptional repression of E-cadherin is related to the E-box motif (5′-CACCTG-3′) of the E-cadherin promoter region to which the transcriptional repressors bind [35].

To elucidate the mechanism of the altered expression of E-cadherin in SMG or overloaded conditions, the RNA levels of Snail, Twist and ZEB1 were determined by qRT-PCR after SMG or overloading treatments. We found that the expression of Snail, Twist and ZEB1 were all largely increased in 24-, 48-, and 72-h treatment of SMG. On the contrary, the expression pattern of Snail, Twist and ZEB1 were quite different. The down expression of Snail was observed in all the time point after OL treatment as compared to the control condition. While the expression of Twist, and ZEB1 were increased at 24-h OL treatment, while reduced gradually at 48- and 72-h OL treatment. Collectively these results suggested the possible involvement of Snail, Twist and ZEB1 in the down regulation of E-cadherin during the SMG. As the time point of increased expression of Snail was consistent to the down regulation of E-cadherin at 24-h SMG treatment, the increased expression of Snail = mainly correlated with the down-regulation of E-cadherin in SMG treatment. However, the roles of Twist and ZEB1 in the down-regulation of E-cadherin during SMG conditions are waiting to be elucidated.

In the condition of normal gravity, loss of cell-cell adhesion was accompanied by the increase of cell-ECM interaction when cells undergoing epithelial-mesenchymal transition [36]. However, after treatment of cells in SMG for three days, both the impaired cell-cell adhesion characterized by the down-regulation of E-cadherin and cell-ECM adhesion characterized by reduced expression of integrin-β1 were observed. These unusual phenotypes may be explained by the specific cellular response to abnormal mechanical stress under the SMG condition. In multipotent mesenchymal stromal cells (MMSC) cells, the expression of integrin-β1 was down-regulated along with integrin-α11, and integrin-αV [37], although the expression of E-cadherin was not detected under SMG in that publication. Therefore, it seems that the down-regulation of E-cadherin and Integrin-β1 at the condition of SMG was not a cell type-specific mechanism.

## 4. Material and Methods

### 4.1. Cultures of HUVEC and MCF-7 Cells

HUVEC and MCF-7 cell lines were used for the present study and were all purchased from ATCC. HUVEC and MCF-7 cells were cultured in DMEM (Invitrogen, Shanghai, China) with 20% (*v*/*v*) fetal calf serum (FBS, Gibco-BRL, Invitrogen, Shanghai, China).

### 4.2. Cell Culture in Rotating Wall Vessel Bioreactor

Clinostat is wildly used for cell culturing, tissue engineering, and space biology research. Rotating Wall Vessel (2D-RWVS) bioreactor was used to create different culture conditions for this study, and it was originally developed by China Astronaut Research and Training Center.

This two-directional, multi-sample cell experimental device can be used to investigate the effect of simulated microgravity effect on the ground [38]. The cells are seeded over a coverslip (22 × 26 × 0.5 mm) and cultured on the slip for 24 h. The slip was then transferred into the bioreactor of 0.04 m in diameter filled with growth medium. According to the equipment manual and reference, the samples were rotated around the horizontal axis at 30 rpm [38]. The cells cannot respond to any change in the rotational direction at this speed. So, this is considered to be the effect of simulated microgravity effect (SMG group). The slip was placed perpendicular to the ground, and the samples were rotated around the vertical axis, could simulate mechanical overloading (OL group). According to the formula a = ω^2^r, it was calculated that average centrifugal acceleration could reach to 0.2 *g* at 30 rpm (*r* = 0.02 m). The cells cultured in the RWVS bioreactor without rotation are considered as the untreated control static control group.

### 4.3. Analysis of Cell Adhesion by Trypsin Digestion and Cell Counting

After the cells were treated under simulated microgravity effect (SMG) or mechanical overloading (OL) for 24, 48, and 72 h, the coverslips were treated with 0.1% trypsin 100 μL and digested for 1 min. The culture plates were dropped from 10 cm high, and then the cells were washed off. The number of exfoliation cells was recorded and analyzed with Chi-square test.

### 4.4. Immunofluorescence Analysis

The cells were kept fixed at room temperature with 4% PFA (Gibico, Invitrogen, Shanghai, China, US origin) for 15 min, permeabilized in 1% (*v*/*v*) TritonX-100 (AMERSCO, Washington, WA, USA) for 10 min, and blocked with 5% BSA (Sigma, USA) for 60 min. For cell adhesion proteins observations, cells were incubated overnight at 4 °C with mouse anti-integrin β1, anti-Paxillin or anti-E-cadherin antibody (1:1000, Sigma, USA) diluted with PBS, and they were incubated at 37 °C for 1.5 h with FITC-conjugated goat anti-mouse IgG (1:100, Zhongshan Biotechnology Co.Ltd., Beijng, China) diluted with PBS. Between those steps, cells are washed three times with PBS. The cells were stained with DAPI (Sigma, USA) to visualize nuclei (blue), mounted in 90% (*v*/*v*) glycerol, and analyzed with a confocal microscope.

The expression of adhesion protein was observed by immunofluorescence after 24, 48, and 72 h, and the relative fluorescence intensity was analyzed by ImageJ software(//imagej.nih.gov/ij/).

### 4.5. Analysis of Cell Adhesion Proteins Expression by Western Blot

Cells in the logarithmic stage of growth were treated with rotating wall vessel bioreactors described above. The total protein of the treated or control cells was extracted with RIPA buffer (150 mM sodium chloride, 1% Triton X-100, 0.5% sodium deoxycholate, 0.1% sodium dodecyl sulphate, 50 mM Tris, pH 8.0). Aliquots of the protein were separated with 12% SDS-PAGE and transferred onto PVDF membranes. The membranes were blocked with PBS-T containing 5% milk, and then incubated overnight at 4 °C with primary antibody (integrin β1, Paxillin or E-cadherin purchased from Sigma), and they were incubated at 37 °C for 1 h with HRP-conjugated secondary antibody. ECL Western blotting analysis system (GE Amersham Biosciences) was used to detect the substrates. The GAPDH was used as the internal control. The relative expression values of detected protein were normalized to the amount of GAPDH. The amount of proteins was assessed by semi-quantitative Western blots densitometry analysis.

### 4.6. Total RNA Extraction and Real-Time PCR

Total RNA was extracted using Trizol. After the RNA was obtained, absorbance was measured at 260 and 280 nm to determine the concentration and purity. 1 µg of total RNA was used for reverse transcription. The cDNA generated by PrimeScriptTM RT reagent Kit with gDNAEraer (Perfect Real Time, Takara, china, cat# RR047A) according to the manufacturer’s instructions.

The ABI 7500 sequence detection system was used (Applied Biosystem) for real-time PCR detection. The sequence of the PCR primers used is as follows:

Snail, forward, 5′-CTCTCTGAGGCCAAGGATCTC-3′:

              reverse,5′-GACATCTGAGTGGGTCTGGA-3′;

Twist, forward, 5′-CATGTCCGCGTCCCACTA-3′;

              reverse, 5′-CTGGGAATCACTGTCCACGG-3′;

ZEB1, forward, 5′-CTTAAGAGCGCTAGCTGCCA-3′;

              reverse, 5′-CGCATTTTCTTTTTGGGCGG-3′;

GAPDH, forward, 5′- AACAGCCTCAAGATCATCAGC-3′;

              Reverse, 5′- GGATGATGTTCTGGAGAGCC-3′;

TaqMan cycle number (Ct) was normalized into relative using 2∆∆*C*t method.

## 5. Conclusions

It can be seen that the expressions of adhesion proteins can be regulated by stress changes and then influence cell adhesion states. EMT transcription factors Snail, Twist, and ZEB1 were up-regulated under SMG, while down-regulated under OL. The regulatory mechanism needs to be further studied.

## Supplementary Materials

Supplementary materials can be found at https://www.mdpi.com/1422-0067/21/4/1349/s1.

## Abbreviations

SMG	simulated microgravity effect
OL	overloading
ECM	extracellular matrix
kDA	kilodalton
DAPI	4′,6-diamidine-2-phenylindol
RWVS	Rotating Wall Vessel
PFA	paraformaldehyde
PBS	phosphate buffer saline
RIPA	Radio Immunoprecipitation Assay
HRP	Horseradish Peroxidase
HUVEC	Human Umbilical Vein Endothelial Cells
MCF-7	Michigan Cancer Foundation - 7
