# Supplementary material for: EMT Transcription Factors Are Involved in the Altered Cell Adhesion under Simulated Microgravity Effect or Overloading by Regulation of E-cadherin
Source: Int J Mol Sci. 2020 Feb 17;21(4):1349. doi: 10.3390/ijms21041349 (PMC7072942; doi:10.3390/ijms21041349)
Supplement: Supplementary file 1 [file ijms-21-01349-s001.pdf]

Table S1. HUVEC Cell exfoliation rate after mechanical treatment

Time	Treatment	Cell number actual value (theoretical value) $\times10^4$			Result
Time	Treatment	Unexfoliated cells	Exfoliated cells	Total	Result
24h	Control	6.25（6.22）	0.75（0.78）	7.00	*χ2 = 3.73848388* *P* = 0.154240541
	SMG	9.67（11.48）	3.25（1.43）	12.92	
	OL	26.75（24.96）	1.33（3.12）	28.08	
	Total	42.67	5.33	48	
48h	Control	34.50（31.99）	2.75（5.26）	37.25	χ^2 = 15.46214985 *P* = 0.000438972
	SMG	21.00（27.26）	10.75（4.48）	31.75	
	OL	29.67（25.91）	0.50（4.25 ）	30.17	
	Total	85.17	14.00	99.17	
72h	Control	69.08（63.99）	5.83（10.92）	74.91	*χ2 = 92.25000581* *P* = 9.29321E-21
	SMG	24.17（45.84）	29.50（7.83）	53.67	
	OL	114.67（98.09）	0.17（16.75）	114.84	
	Total	207.92	35.50	234.42	

Table S2 MCF-7 Cell exfoliation rate after mechanical treatment

Time	Treatment	Cell number actual value (theoretical value) $\times10^4$			Result
Time	Treatment	Unexfoliated cells	Exfoliated cells	Total	Result
24h	Control	6.83 (6.61)	0.58 (0.81)	7.41	*x^2 = 4.363168461* *P = 0.112862589*
	SMG	8.83 (10.78)	3.25 (1.31)	12.08	
	OL	26.75 (25.03)	1.33 (3.05)	28.08	
	Total	42.41	5.16	47.57	
48h	Control	34.50（32.89）	2.75（4.36）	37.25	*x^2 = 11.45367974* *P* = 0.003257355
	SMG	18.83 (23.47)	7.75 (3.11)	26.58	
	OL	29.67（26.64）	0.50（3.53）	30.17	
	Total	83.00	11.00	94.00	
72h	Control	69.08（71.65）	5.83（3.25）	74.91	*x^2* = 261.3459895 *P* = 1.77599E-57
	SMG	24.50（51.33）	29.17（2.33）	53.67	

OL	114.67（98.09）	0.17（16.75）	114.84
Total	207.92	35.50	234.42

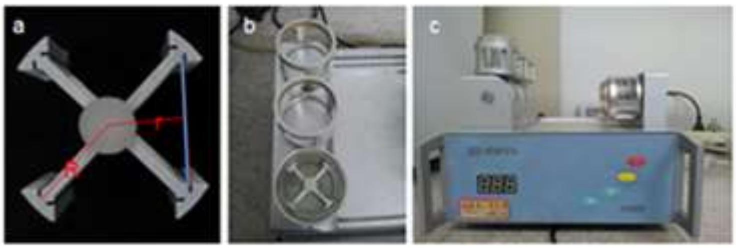

Figure S1. The experimental setup. a.Cell culture frame (the blue line indicate slip with cells). b. The culture bottle is fixed on the equipment. c.The equipment of 2D-RWVs. The culture bottle is fixed on the equipment and rotates around the horizontal axis (right) to simulate microgravity effect. The culture bottle is fixed on the equipment and rotates around the vertical axis (left) to obtain mechanical overloading.
